# Protection of the transplant kidney during cold perfusion with doxycycline: proteomic analysis in a rat model

**DOI:** 10.1186/s12953-020-00159-3

**Published:** 2020-04-20

**Authors:** Michael A. J. Moser, Katherine Sawicka, Jolanta Sawicka, Aleksandra Franczak, Alejandro Cohen, Iwona Bil-Lula, Grzegorz Sawicki

**Affiliations:** 1 Department of Surgery, University of Saskatchewan, Saskatoon, Saskatchewan Canada; 2 Department of Anatomy, Physiology and Pharmacology, University of Saskatchewan, Saskatoon, 107 Wiggins Road, Saskatoon, Saskatchewan S7N 5E5 Canada; 3 Department of Medical Laboratory Diagnostics, Division of Clinical Chemistry, Wroclaw Medical University, Wroclaw, Poland; 4 Proteomics and Mass Spectrometry Core Facility. Life Sciences Research Institute, Dalhousie University, Halifax, Nova Scotia Canada

**Keywords:** Cold perfusion, Doxycycline, Kidney proteome, Kidney transplantation, Pharmacological protection

## Abstract

**Background:**

It has been previously shown that doxycycline (Doxy) protects the kidney from preservation injury by inhibition of matrix metalloproteinase. However, the precise molecular mechanism involved in this protection from injury is not known. We used a pharmaco-proteomics approach to identify potential molecular targets associated with kidney preservation injury.

**Methods:**

Rat kidneys were cold perfused with or without doxycycline (Doxy) for 22 h. Kidneys perfusates were analyzed for the presence of injury markers such as lactate dehydrogenase (LDH), and neutrophil-gelatinase associated lipocalin (NGAL). Proteins extracted from kidney tissue were analyzed by 2-dimensional gel electrophoresis. Proteins of interest were identified by mass spectrometry.

**Results:**

Triosephosphate isomerase, PGM, dihydropteridine reductase-2, pyridine nucleotide-disulfide oxidoreductase, phosphotriesterase-related protein, and aminoacylase-1A were not affected by cold perfusion. Perfusion with Doxy increased their levels. N(G),N(G)-dimethylarginine dimethylaminohydrolase and phosphoglycerate kinase 1 were decreased after cold perfusion. Perfusion with Doxy led to an increase in their levels.

**Conclusions:**

This study revealed specific metabolic enzymes involved in preservation injury and in the mechanism whereby Doxy protects the kidney against injury during cold perfusion.

## Background

End stage renal disease (ESRD) is the final phase of chronic kidney disease. Hemo- or peritoneal dialysis is life-saving in patients who progress to chronic renal failure [1]. However, since dialysis is accompanied by low quality of life and life expectancy, kidney transplantation, as a form of renal replacement therapy, is the option of choice for many patients [2]. In North America, approximately 75% of all solid organ transplants performed are kidney transplants [3].

The transplantation of a kidney or any other organ from one person to another is necessarily accompanied by injury, whether at the time of procurement, the preservation time, or during the completion of the anastomosis. Injury may occur during those periods of either warm [4] or cold [5] ischemia.

The consequences include delayed allograft function (DGF), acute tubular necrosis, and acute kidney injury (AKI) [6]. Microscopic injury can also contribute to infarction and to an increased incidence of acute rejection in the early transplant period [5]. There are numerous factors associated with worse injury during transplantation, including non-optimal preservation, [7] hypoxia, [4] hypo- or normothermic conditions [8] and perfusion method [7, 9, 10].

There exist a number of kidney preservation solutions, yet in spite of this, more than 30% of transplant kidneys from donation after circulatory death donors have delayed function after transplantation [11].

In this study, we focused on the changes observed in the proteome of kidney subjected to ischemia during machine cold perfusion. The oxidative stress of the graft during ischemia and reperfusion leads to an increased activity of matrix metalloproteinases (MMPs) [12, 13], and MMPs, in turn, are able to degrade intra- and extracellular proteins [14] (causing the changes of protein content in kidneys) leading to organ injury and dysfunction,. Therefore, the main aim of the current study was to better describe the nephroprotective activity of doxycycline (MMPs inhibitor) during ex vivo kidney cold perfusion in a rat model. The identification of the possible mechanisms whereby doxycycline protects the transplant kidney could yield other targets and hence other pharmacologic approaches to protecting the transplant kidney from preservation injury.

## Methods

The experimental procedures described below conform to the Guide to the Care and Use of Experimental Animals published by the Canadian Council on Animal Care and Committee on Animal Care of Polish Academy of Science. The study was approved by Animal Research Ethics Board (University of Saskatchewan, Animal Use Protocol number 20060054) and by the Local Ethics Board of the Institute of Immunology and Experimental Therapy (Polish Academy of Science, Wroclaw, Poland, Animal Use Protocol number 018/2019).

### Rat kidney model

**Fig. 1:**
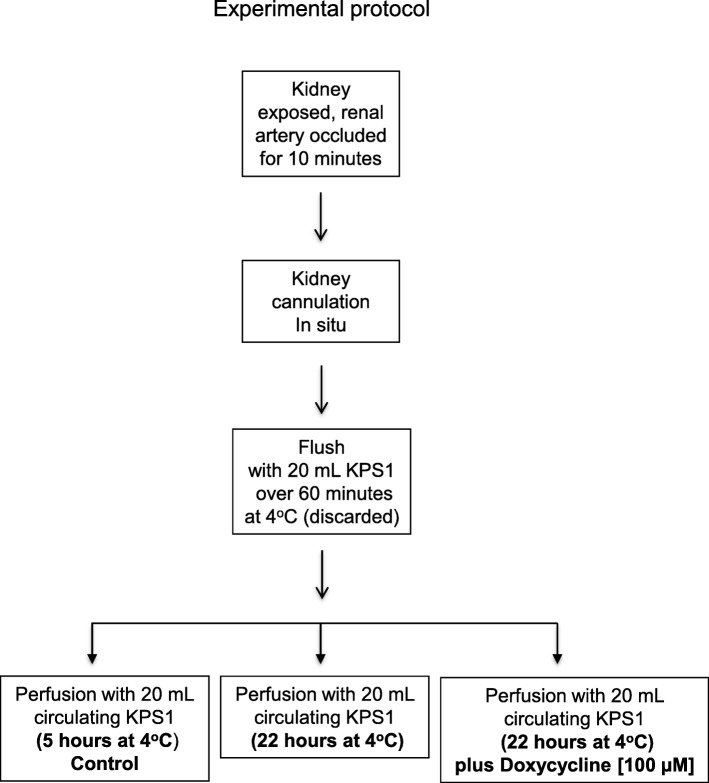
Experimental protocol for perfusion of rat kidney with and without doxycycline. KPS1 – Kidney Perfusion Solution 1

The experimental protocol for perfusion of rat kidney is shown in Fig. 1. Male Sprague-Dawley rats (200–250 g, Charles River, Burlington, Canada) (4 rat/group) were anesthetized with isoflurane. The left renal artery was ligated in situ for 10 min of warm ischemia, then cannulated and the kidney was removed and rapidly cooled to 4 °C. The kidney and tubing were connected to the perfusion apparatus and flushed over 60 min with 20 mL of KPS-1 (kidney perfusion solution-1, Organ Recovery, Chicago, USA).

The machine cold perfusion apparatus consisted of a micro-pump with flow rate of 0.5 mL/min, operating in a 4 °C cold room. The KPS-1 (20 mL with or without 100 μM Doxycycline) was continuously infused via the renal artery and the effluent passively drained via the renal vein to be recirculated via the pump.

The kidney was perfused for 22 h; this time was longer than the usual clinically used preservation time in order to increase the chances of observing significant injury of the rat kidney. Perfusate samples of 0.5 mL were collected after 22 h and stored at − 80 °C. At the end of the perfusion, the kidney was stored at − 80 °C.

### Preparation of kidney protein extracts

For immunoblot studies, frozen kidney tissue was homogenized on ice in 150 mM NaCl, 50 mM Tris-HCl (pH 7.4) containing protease inhibitor cocktail (Sigma, St Louis, MO, USA) and 0.1% Triton X-100. Homogenates were centrifuged at 10000 g at 4 °C for 10 min, and the supernatant was stored at − 80 °C until further use.

Protein samples for 2DE were prepared at room temperature by mixing frozen (− 80 °C), powdered kidney tissue (40 to 60 mg wet weight) with 200 μL rehydration buffer (8 mol/L urea, 4% CHAPS, 10 mmol/L DTT, 0.2% Bio-Lytes 3/10 [BioRad]) at room temperature. Samples were sonicated twice for 5 s and centrifuged for 10 min at 10000 x g to remove any insoluble particles [15].

### Measurement of neutrophil gelatinase-associated lipocalin (NGAL) level

NGAL concentration in perfusates was measured using Lipocalin-2 (NGAL) Rat ELISA Kit (Abcam, ab119602) according to manufacturer’s instruction. The concentration of NGAL was proportional to the intensity of the colored reaction product (450 nm). All samples (*n* = 4/group) were analyzed in duplicate. NGAL level was expressed as ng of protein per ml of perfusate.

### Measurement of lactase dehydrogenase (LDH) activity

The activity of LDH in perfusates was measured with the LDH Activity Assay kit (Sigma-Aldrich, Billerica, MA, US, Reference number: MAK 066) according to the manufacturer’s instruction. The LDH activity was measured as the amount of NADH generated within a minute from the conversion of lactate into pyruvate by LDH, which was detected by colorimetric assay at 450 nm The LDH activity was expressed as milliunits per milliliter of perfusate (mU/ml which is nmol of NADH/min per milliliter of perfusate). All samples (*n* = 4/group) were analyzed in duplicate.

### Measurement of protein

Protein concentration in perfusates was evaluated by measurement of absorbance at 220 nm and expressed as A_220nm_ per ml of perfusate. Protein concentration in the kidney extract in homogenized buffer was measured with the Bradford protein assay (Bio-Rad, Hercules, CA, USA).

### Two-dimensional electrophoresis

Protein (0.4 mg) from kidney samples (*n* = 4/group) was applied to each of 11 cm immobilized linear pH gradient (5 to 8) strips (IPG, BioRad), with rehydration for 16–18 h at 20 °C. For isoelectrofocusing, the BioRad Protean IEF cell was used with the following conditions at 20 °C with fast voltage ramping: step 1: 15 min with end voltage at 250 V; step 2: 150 min with end voltage at 8000 V; step 3: 35000 V-hours (approximately 260 min). After IEF, the strips were equilibrated according to the manufacturer’s instructions. Second dimension of 2DE was then carried out with Criterion pre-cast gels (8–16%) (BioRad). After separation, proteins were detected with Coomassie Briliant Blue R250 (BioRad). To minimize variations in resolving proteins during the 2DE run, 12 gels were run simultaneously using a Criterion Dodeca Cell (BioRad). Because of this limitation, for 2DE analysis we used 4 kidneys from each of 3 group. All the gels were stained in the same bath and next scanned with calibrated densitometer GS-800 (BioRad). Quantitative analysis of protein spots intensity from 2DE was measured with PDQuest 8.0.1 measurement software (BioRad). The protein spot sensitivity threshold used to determine significant changes in protein spot size and density is based on 4 parameters: minimum peak value sensitivity, smallest spot area, largest spot area, and a noise filter level. Using these criteria for protein resolution and staining, we are able to obtain high reproducibility to analyze both a single protein from the same sample run in different gels [16] and a specific protein from different samples [17]. Only protein spots with relative intensity between 2 and 500 arbitrary units were considered for analysis. Protein spots, that showed a significant difference between groups, were selected for further analysis.

### Mass spectrometry

Proteins from 2DE were excised from the gel. Prior to digestion, gel bands were destained, by placing the gel spots/bands in a centrifuge tube containing 1 ml of a 50% methanol, 5% acetic acid solution, and shaking for approximately 1 h, or until destaining was achieved. After destaining, gel bands were rinsed with 1 ml of water. Then, the gel pieces containing protein of interest were processed a robotic protein with digesting system (Investigator ProGest, Genomic Solutions, Huntingdon, UK). Samples were reduced using 100uL of 10 mM DTT, alkylated with 100 μL of 55 mM iodoacetamide and finally digested overnight with trypsin (Promega Trypsin Gold, Mass Spectrometry Grade. Promega, PN V5280) at 37 °C.The peptides were extracted from the gel pieces by three 20 min incubations with a solution (30 μL) containing acetonitrile (50%) and formic acid (5%) in LC − MS-grade water with gentle agitation. The extracts were pooled and dried using a vacuum concentrator (Speed Vac Concentrator, SPD 111 V-230, Thermo Electron Corp. Gormley, Canada) and finally resuspended in LC − MS-grade water (15 μL) containing acetonitrile (3%) and formic acid (0.5%). LC-MS/MS was performed using a nano flow liquid chromatography system (Ultimate3000RSLCnanno, ThermoScientific, Toronto, Canada) interfaced to a hybrid ion trap-orbitrap high-resolution tandem mass spectrometer (VelosPro, ThermoScientific, Toronto, Canada) operated in data-dependent acquisition (DDA) mode. One μl of each sample was injected onto a capillary column (50 cm × 75 μm PicoTip/PicoFrit Self packed column with Jupiter C18 4u chromatographic media, Phenomenex, Torrance, Ca, USA) at a flow rate of 300 nl x min^− 1^. Samples electro-sprayed at 1.2 kV using a dynamic nanospray probe. Chromatographic separation was carried out using 90 min linear gradients (Mobile Phase A: 0.1% formic acid in MS-grade water, mobile phase B: 0.1% formic acid in MS-grade acetonitrile,) from 3% B to 35% B over 60 min, then increasing to 95% B over 5 min. MS/MS spectra were acquired using both collision-induced dissociation (CID) and higher-energy collisional dissociation (HCD) for the top 15 peaks in the survey 30,000 resolution MS scan. The raw files were acquired (Xcalibur, ThermoFisher) and exported to Proteome Discoverer 2.2 (ThermoFisher, Toronto, Canada) software using SequestHT node for peptide and protein identification against the Swiss-Prot databases for rodents. Databases were obtained from Uniprot (Uniprot.org). The latest and complete UniprotKB (Swiss-Prot + Unvreviwed TrEMBL) proteome from *Rattus norvegicus* (ID 10116) was downloaded prior to data analysis. The following parameters were selected for the database search: trypsin digest, one missed cleavage, 10 ppm precursor mass tolerance and 10 ppm/0.8 Da for fragment (MSMS) mass tolerance for HCD and CID respectively. Carbamidomethyl cysteine was selected as static modification, and oxidized methionines were set as dynamic modification [17, 18]. Peptide and protein False Discovery Rate (FDR) were set to 0.1% using Percolator [19]. The SEQUEST scoring algorithm [20] was used to justify the accuracy of protein identification, which is incorporated into the search engine algorithm.

### Immunoblot analysis

Protein extracts from the same kidneys that were used for 2DE (30 μg protein) were separated by SDS-PAGE (*n* = 3/group). After electrophoresis proteins were transferred to a PVDF membrane (BioRad). Membranes were probed with rabbit polyclonal antibodies (Abcam) against triosephosphate, aminoacylase 1, rabbit polyclonal anti-MLC2, phosphoglycerate kinase 1 according to the supplier’s instructions. Goat anti-rabbit secondary antibodies, tagged with Alexa Fluor647 (ThermoFisher Scientific, Waltham, MA, USA), were used and membranes developed with VersaDoc5000 using appropriate filters. Band densities were determined with Quantity One software (BioRad). Actin was used as a protein loading control.

### Electron microscopy

Kidneys without cold perfusion, kidneys after 22 h of cold perfusion and kidneys after 22 h cold perfusion with Doxy (*n* = 3/group) were flushed with glutaraldehyde and 1 mm cubes of tissue were excised from the upper pole in each kidney for electron microscopy, post fixed in osmium tetroxide, dehydrated in graded ethanol, and embedded in an epoxy resin. Ultrathin sections were cut and stained with uranyl acetate and lead citrate and random areas photographed using digital transmission electron microscopy (Hitachi H9500, Tokyo, Japan). These were reviewed in blinded fashion by a renal pathologist.

### Statistical analysis

The protein spot levels were analyzed using PDQuest measurement software and evaluated by Kruskal Wallis and Mann-Whitney U test. The proteins of interest (determined by PDQuest to be statistically significant – *p* < 0.05) were also analyzed with unpaired, one-tailed t-test with Welch’s correction, followed by identification by mass spectrometry. ANOVA or Kruskal-Wallis test was used in functional studies. For immunoblot experiments a two-tailed unpaired t-test with Welch’s correction was used. Data are expressed as mean ± SEM.

## Results

### LDH, NGAL and total protein levels as markers of kidney injury

**Fig. 2:**
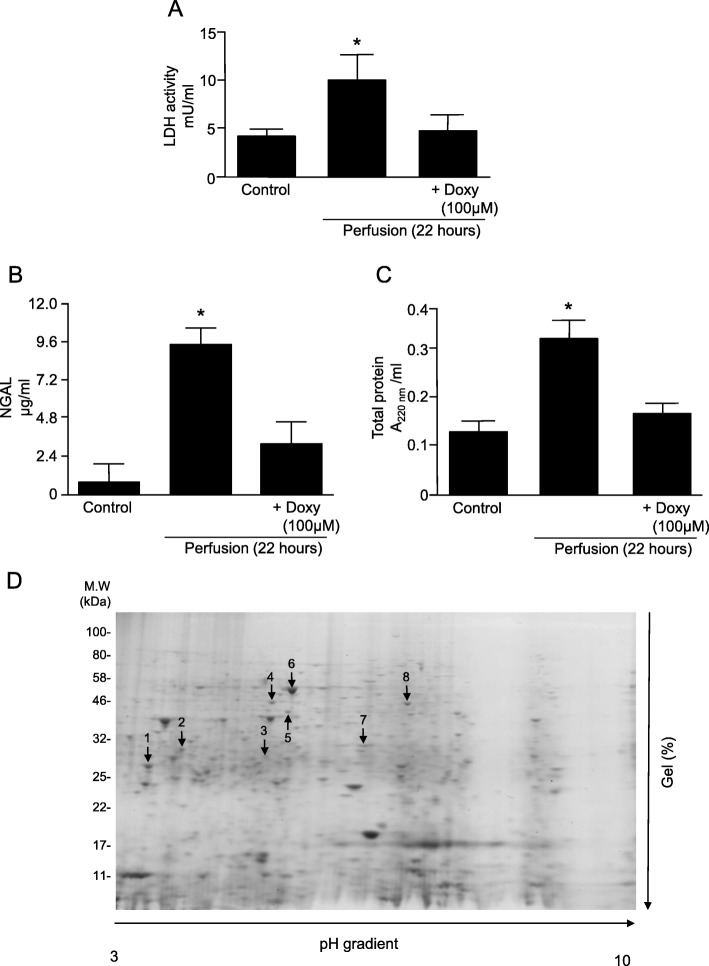
Levels of markers of kidney injury in perfusates. **a** Lactate dehydrogenase (LDH); **b** Neutrophil gelatinase-associated lipocalin (NGAL). **c** Total protein level (**d**) Representative 2DE gel showing protein expression in kidney tissue after cold perfusion. * represents *p* < 0.05 vs Control group, *n* = 4

LDH, NGAL and total protein levels were measured in perfusates as markers of injury. A two-fold increases in LDH activity and total protein level were observed in perfusates from ischemic kidneys perfused without Doxy (Fig. 2a,c), compared to that seen in control kidneys. An almost 10-fold increase in NGAL levels was seen in perfusates from ischemic kidneys compared to the controls (Fig. 2b). Levels of all analyzed markers were normalized by 100 μM Doxy (Fig. 2a-c).

### Evaluation of kidney injury by electron microscopy

**Fig. 3:**
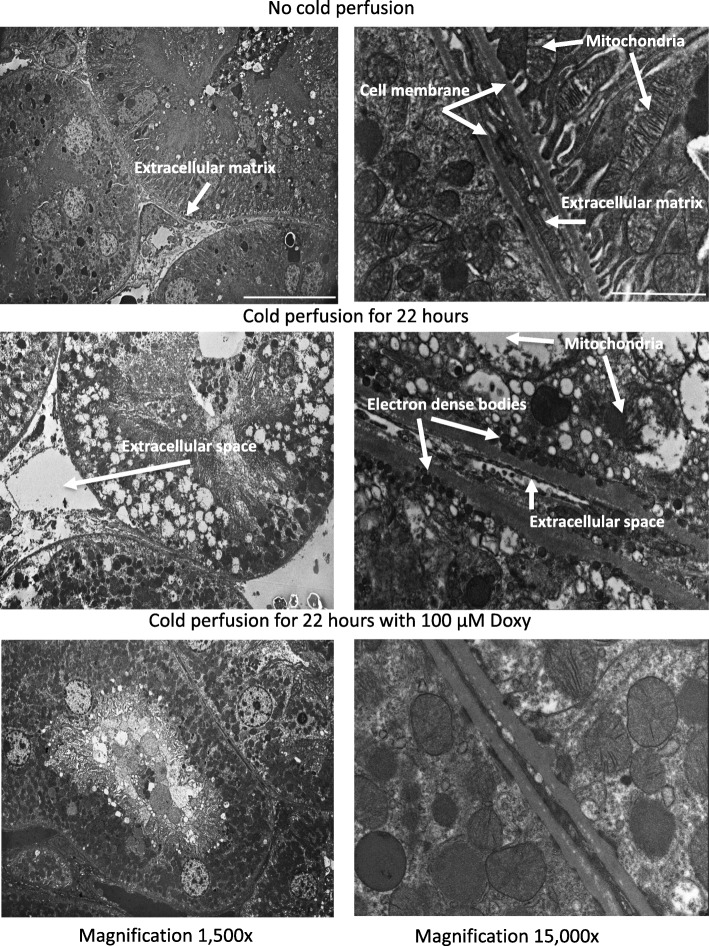
Representative electron micrographs of control rat kidneys (upper micrographs) and perfused without (middle micrographs) or with Doxy (bottom micrographs). Left micrographs are at 1500x magnification, micrographs on right side are at 15000x magnification. Doxy – doxycycline

Because in our previous studies we showed that cold perfusion damages structure of kidney cells [12], the effect of Doxy on kidney cells and subcellular structures during cold perfusion was evaluated by electron microscopy (Fig. 3, Additional file 4).

Cold perfusion caused separation of cells and enlarging of the extracellular space (Fig. 3, middle panels). Perfusion with Doxy protected the kidney from this expansion of extracellular space (Fig. 3, bottom panel). Damaged mitochondria and formation of dense bodies were observed after cold perfusion (Fig. 3, middle panel, magnification 15,000x). Perfusion with 100 μM Doxy protected mitochondria and inhibited formation of dense bodies (Fig. 3, bottom panel, magnification 15,000x).

### Analysis of kidney proteome by 2DE

Analysis of kidney homogenates by 2DE revealed 8 protein spots where a significant difference was seen between any of the three groups (control, 22 h perfusion, and 22 h perfusion with solution containing Doxy) (Fig. 2d). Mass spectrometry analysis identified these protein spots as: triosephosphate isomerase (TPI), phosphoglycerate mutase (PGM), dihydropteridine reductase-2, pyridine nucleotide-disulfide oxidoreductase, phosphotriesterase-related protein, aminoacylase-1A, N(G),N(G)-dimethylarginine dimethylaminohydrolase, and phosphoglycerate kinase 1 (Table 1). The sequences of identified peptides are provided in Additional file 1.

**Table 1:** Identification of protein spots

Protein spot No.	Score SEQUEST	Queries matched	Sequence coverage (%)	pI (Exp^a^)/MW (Exp) (kDa)	Identified protein^b^ (UniProtKB/Swiss-Prot ID)
1	98.53	10	51	6.81(4.05)/26.85(24)	Triosephosphate isomerase (P48500)
2	155.96	13	56	6.73(4.46)/28.83 (26)	Phosphoglycerate mutase (P25113)
3	67.64	8	51	7.37(5.60)/25.55 (27)	Dihydropteridine reductase-2 (P11348)
4	186.56	14	31	5.25(5.76)/55.49(37)	Pyridine nucleotide-disulfide oxidoreductase (Q68FT6)
5	98.45	12	46	6.47(5.91)/39.14 (33)	Phosphotriesterase-related protein (Q63530)
6	595.06	29	76	6.11(6.00)/45.80(45)	Aminoacylase-1A (Q6AYS7)
7	68.57	8	38	5.81(6.90)/31.42(27)	N(G),N(G)-dimethylarginine dimethylaminohydrolase (O08557)
8	184.72	17	52	7.56(7.40)/44.54(40)	Phosphoglycerate kinase 1 (P16617)

^a)^ Exp, experimental

^b)^ List of peptides in Additional file 1

**Fig. 4:**
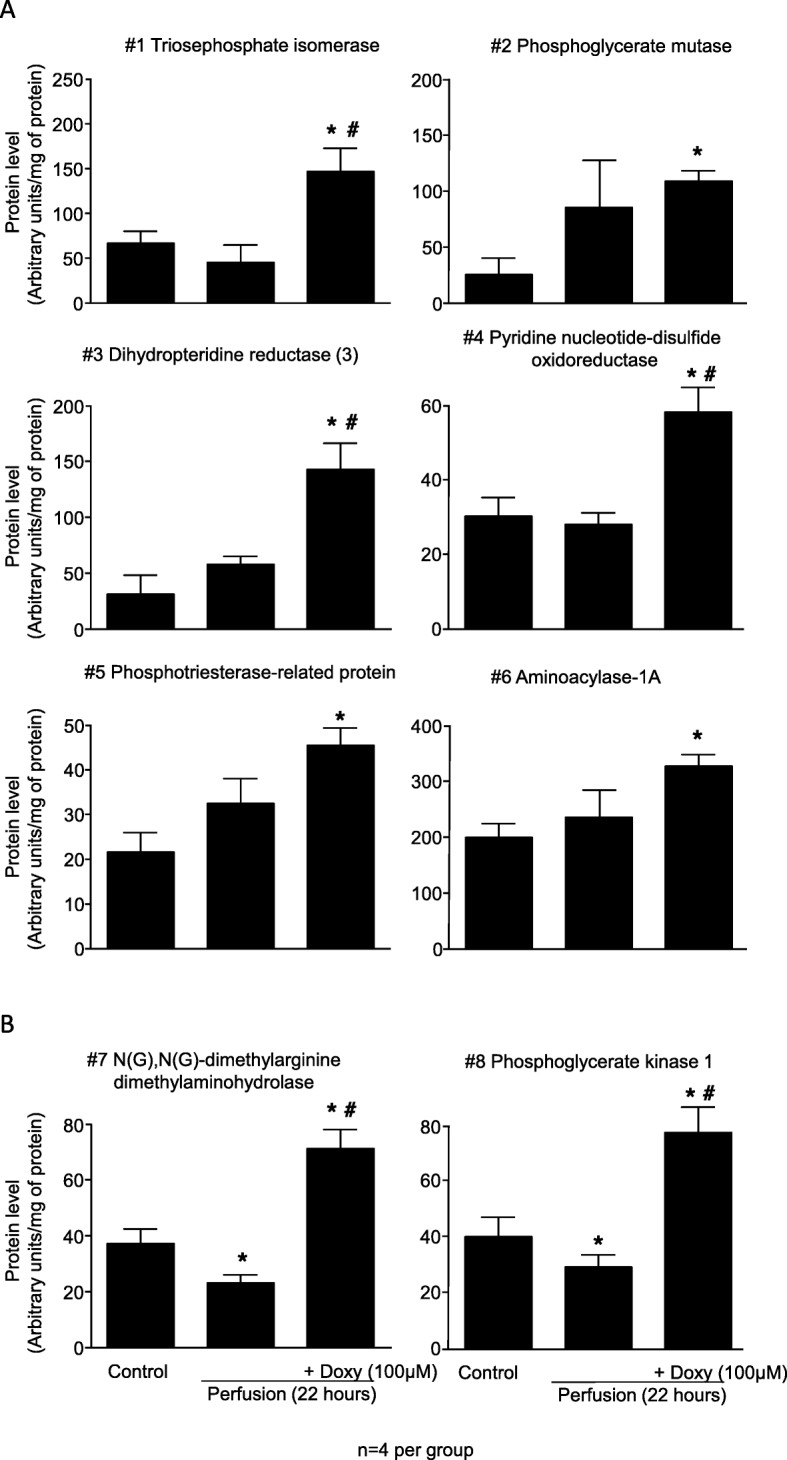
Results of densitometric analysis of protein spots from 2DE gels. **a** Shows protein increased by Doxy but not affected by cold perfusion. **b** Shows proteins affected by cold perfusion (decreased level) and by Doxy (increased level). Doxy – doxycycline* represents *p* < 0.05 vs Control group, # *p* < 0.05 vs 22 h perfusion group, n = 4

Levels of the 6 first proteins (1–6, Table 1) were not affected by cold perfusion, however 100 μM Doxy significantly increased levels of these proteins compared to control (Fig. 4a). Levels of protein 1, 3 and 4 were significantly higher when perfused with Doxy compared to those perfused without Doxy (Fig. 4a). Levels of protein 7 and 8 (Table 1) were decreased after cold perfusion (Fig. 4b). Perfusion with Doxy increased the level of these protein by aproximately two fold in comparison to controls and kidneys perfused without Doxy (Fig. 4b). Full size 2DE gel images are provided in Additional file 2.

### Immunoblot analysis of some identified proteins

**Fig. 5:**
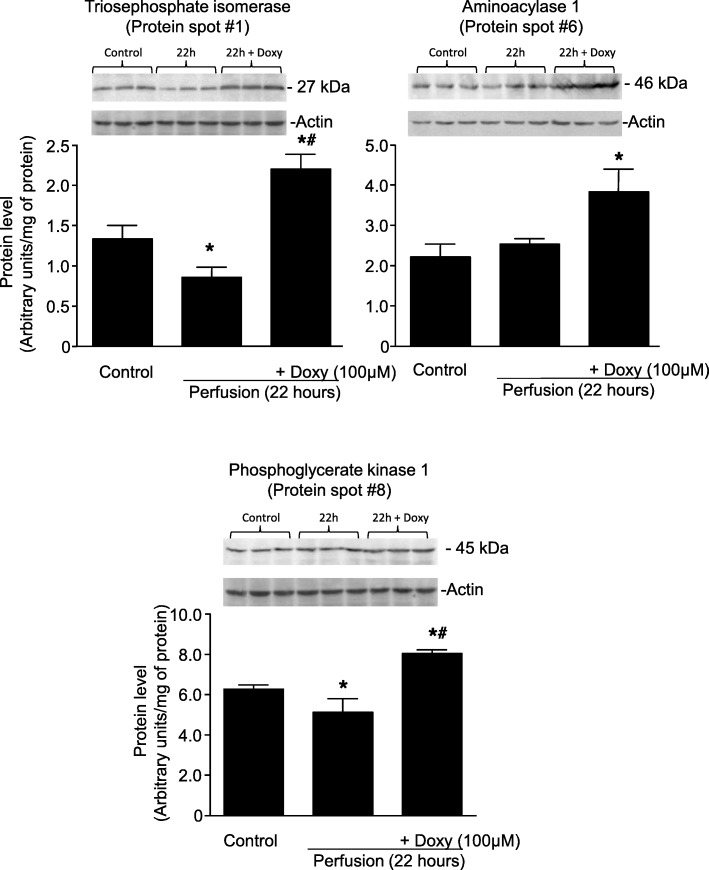
Verification of results from 2DE by analysis of changes of protein levels with immunoblotting. Doxy – doxycycline, * represents *p* < 0.05 vs Control group, # *p* < 0.05 vs 22 h perfusion group, *n* = 3

Protein level changes observed from 2DE for some proteins, was verified by immunoblotting (according to commercial availability of antibodies). In 2DE triosephosphate isomerase (protein #1), aminoacylase 1 (protein #6) were not changed in 22 h perfusates (Fig. 4a). Phosphoglycerate kinase (protein #8) was identified as significantly decreased in the perfusate at 22 h. In perfusates from kidneys protected with Doxy levels of these 3 proteins were significantly increased in comparison to control levels (Fig. 5a and b).

The results of immunoblot analysis were in accordance with the changes in protein levels observed in 2DE for protein #6 (aminoacylase-1) and protein #8 (phosphoglycerate kinase). For protein #1 (triosephosphate isomerase) a significant decrease in its level after 22 h of perfusion compared to control group was observed in WB analysis (Fig. 5), whereas there was no difference between those groups in 2DE (see Fig. 4a). Full size Western blots are provided in Additional file 3.

## Discussion

The preservation of kidneys for transplantation relies mainly on hypothermia, usually at a temperature of 4 °C, to decrease cellular metabolism and conserve stores of adenosine triphosphate. Because metabolism is ongoing, albeit at a slower rate, the duration of cold ischemia should be minimized as much as possible [8], especially in donors over 55 year old or marginal donors [2]. Even with machine cold perfusion, significant preservation injury nonetheless occurs, and likely contributes to delayed graft function (DGF) and acute tubular necrosis in the transplanted kidney [21]. Injury of any cause is suspected to lead to a decrease in kidney function, shortened graft survival, and an increase in rejection due to increased activation of the immune system [4, 22, 23].

In our previous studies we also showed that both hearts [13] and kidneys [12] subjected to ischemia showed an increased activity of matrix metalloproteinase-2. Furthermore, we showed that inhibition of MMP activity by pharmacological agents protects kidneys from cold perfusion injury. MMPs therefore likely contribute to the injury cascade that is involved in preservation injury.

In the current study, 22 h of machine cold perfusion led to significant kidney injury, as shown by release of LDH (cells injury marker) and NGAL (marker of tubular epithelial cell damage [24]). Cold perfusion with standard preservation solution including doxycycline, an inhibitor of MMPs, led to decreased kidney injury, as previously described [12].

MMPs are zinc-containing enzymes initially characterized as mainly functioning to degrade extracellular matrix proteins. Further study has shown that they have a multitude of other functions including cellular proliferation, angiogenesis, and inflammation. They have also been shown to have intracellular effects on metabolism, including effects on mitochondrial function [25], and have been shown to reside in mitochondrial membranes [26]. MMP-2 and MMP-9 has been shown to damage mitochondrial DNA, chaperone machinery, and increasing mitochondrial membrane permeability, leading in turn to mitochondrial dysfunction and ROS generation [27–30]. MMPs are known to be activated by oxidative stress, other proteases, and HSP70 [31], so it seems logical that they are part of the mechanism of injury of preserved organs.

We sought to investigate the specific mechanism by which inhibition of MMPs protects kidneys from cold preservation injury. We assessed the proteome changes in kidneys subjected to machine cold perfusion in comparison to those perfused with a solution containing Doxy. 2DE analysis of kidney tissue showed a significant difference in 8 enzymes, all involved in cellular and mitochondrial metabolism. These include 3 involved in glycolysis (triosephosphate isomerase [TPI], phosphoglycerate kinase 1 [PK-1], and phosphoglycerate mutase [PGM]), one involved in the urea cycle (aminoacylase-1A), one that regulates nitric oxide synthesis (N(G),N(G)-dimethylarginine dimethylaminohydrolase) and others (dihydropteridine reductase-2, pyridine nucleotide-disulfide oxidoreductase, phosphotriesterase-related protein). For all 8 of these proteins, perfusion with Doxy led to an increase in protein expression compared to the pre-cold-perfusion state.

Of particular interest are TPI, PK-1, and N(G),N(G)-dimethylarginine dimethylaminohydrolase, which were decreased by cold preservation and for which perfusion with Doxy led to a ‘correction’ of these decreases. TPI is a key enzyme in glycolysis and in the provision of substrates for cellular respiration and energy production in the mitochondria. N(G),N(G)-dimethylarginine dimethylaminohydrolase, also known as asymmetric dimethylarginine (ADMA), is thought to reside primarily in the mitochondria and plays a role in regulating the expression of nitric oxide, which has many functions including vasodilation, inflammation, and maintenance of the endothelium [32]. PK-1 is also a key enzyme in both glycolysis and gluconeogenesis, it is somewhat unique in that it activates enzymes in both directions of these opposing pathways [33].

These findings are in keeping with the electron micrograph findings, and together they suggest that inhibiting MMPs protects the transplant kidney by actions on the mitochondria in addition to the more well-known mechanism of protecting the extracellular matrices. Whether the preservation of mitochondria is by maintenance of mitochondrial function, or by protecting against structural changes to the mitochondria by inhibiting the MMPs within, remains to be elucidated.

In a prior study, we documented the differences in proteins released into the perfusate during machine cold perfusion [34]. This revealed very different proteins alpha-1-antitrypsin, peroxiredoxin-2, and neutrophil gelatinase-associated lipocalin (NGAL - a standard marker of renal injury) being released during preservation injury. There was no pharmacologic protection arm in that study. The current study is different in that we documented intracellular proteins and we were able to document changes in proteins that are involved in the cell’s metabolism via glycolysis, the urea cycle, and nitric oxide synthesis, and the differences in kidneys protected and not protected by doxycycline.

Other teams and collaborators are looking at the addition of hydrogen sulfide and carbon monoxide to the cold perfusion solution as means of protecting the transplant kidney from injury [35, 36]. The mechanism for both approaches appears to be through the induction of a ‘hibernation-like-state’, that is, reducing the metabolism so that ATP and other energy stores are preserved. Our work, on the other hand, suggests that inhibition of MMPs protects the kidneys by a different mechanism, that of maintenance of mitochondrial function and, in turn, structure.

Mitochondria produce ATP to fuel essential cellular processes, but on the other hand, they can contribute to cell death and in turn organ failure by production of ROS [37]. It is understandable that maintaining of their homeostasis has a crucial role in cell-survival.

The preservation of mitochondrial structure and their ability to balance ATP production and consumption in hypothermic conditions is one of the factors that allows hibernating animals to survive the hibernation season. The described phenomenon can be the explanation for their resistance to IRI resembled by repetitive cycles of torpor and arousal during hibernation state [38, 39].

Hendriks et al. showed that lowering renal temperature progressively favors mitochondrial ROS production over mitochondrial respiration in human kidney cells [39]. Interestingly, renal cells of hibernating animal (hamster) undergoing hypothermia and rewarming showed not only the maintenance of ATP production and a proper mitochondrial network structure, but also no increase in ROS [37].

In addition to the fact that hamster kidney cells had more potent oxidative phosphorylation (and thus the larger spare oxidative capacity) than human kidney cells, hamster cells also showed adaptation to anaerobic ATP production (via glycolysis). The results suggested that glycolysis contributes to maintaining ATP synthesis during cold ischemia in hibernating animals [37].

Our results indicating the potential increase in glycolysis are consistent with the metabolic adaptations observed in hibernating animals. However, it is important to remember that increased level of metabolic enzymes does not necessarily mean an increase in the enzymatic reaction, and further studies are needed to confirm this conceptual reasoning.

Our results are also in accordance with the study showing that Doxy increased expression of genes involved in glycolysis in multiple human cell lines [40]. However, the mechanism behind Doxy-induced switch from oxidative metabolism towards glycolysis is not fully elucidated. It has been suggested it can be an adaptive mechanism to decreased oxidative phosphorylation (resulting from disturbance of mitochondrial proteostasis through Doxy effect on mitochondrial translation) [40].

Doxy can contribute to protection of mitochondria against ischemic damage in different ways. Except for its potential involvement in energy metabolism, it can inhibit MMP activity (by chelating zinc ion in catalytic site of MMPs [41]), as well as Doxy can directly scavenge ROS – superoxide [42].

Currently, doxycycline is the only clinically approved inhibitor of MMPs. Thus, although non-selective, it is the most fitting for this research, providing the fastest way for the potential future clinical trial. To the best of our knowledge, Doxy has not been tested in clinical prevention of organs during transplantation yet. However, Doxy (submicrobial doses) has been shown to significantly reduce cardiac MMP-2 activity in the clinical trial in patients undergoing artery bypass graft surgery with cardiopulmonary bypass (cardiac ischemia/reperfusion injury) [43].

The processes related to renal injury during transplantation is multifactorial, and it requires complex approach. As combinations of carbon monoxide releasing molecules and doxycycline and H_2_S are being contemplated, it will be important to document which components of metabolism are being targeted so as to avoid the effects of one drug cancelling out the effects of the other. It will be interesting to see what happens when we combine the two pharmacologic approaches in upcoming collaborations. The pharmacological approaches addressing any of the pathways of injury related to organ preservation and transplantation (including oxidative stress, energy metabolism and structural changes) should be of interest.

## Conclusions

Matrix metalloproteinases are ubiquitous and are activated by oxidative stress and other proteases. The inhibition of matrix metalloproteinases is able to protect the kidney from cold preservation injury. This study suggests that this may be as a result of mitochondrial protection, whether from the direct effects of MMPs from within or by the maintenance of mitochondrial metabolism and hence the maintenance of mitochondrial structure, in addition to protection of the ECM.

## Supplementary information

**Additional file 1.** The sequences of identified peptides.

**Additional file 2.** Two-dimensional gel electrophoresis gel images.

**Additional file 3.** Western blot images

**Additional file 4.** Electron microscopy

## Data Availability

The sequences of identified peptides, full size 2-DE gel images and western blot images are available in Additional material. The additional data are available from the corresponding author on reasonable request.

## Abbreviations

ADMA: N(G),N(G)-dimethylarginine dimethylaminohydrolase
AKI: Acute kidney injury
DDA: Data-dependent acquisition
DGF: Delayed allograft function
Doxy: Doxycycline
ESRD: End stage renal disease
FDR: False discovery rate
HCD: Higher-energy collisional dissociation
KPS-1: Kidney perfusion solution-1
LDH: Lactate dehydrogenase
MMPs: Matrix metalloproteinases
NGAL: Neutrophil-gelatinase associated lipocalin
PGM: Phosphoglycerate mutase
TMB: Tetramethylbenzidine
TPI: Triosephosphate isomerase
2DE: 2-dimensional electrophoresis
