# Supplementary material for: Protection of the transplant kidney during cold perfusion with doxycycline: proteomic analysis in a rat model
Source: Proteome Sci. 2020 Apr 20;18:3. doi: 10.1186/s12953-020-00159-3 (PMC7171734; doi:10.1186/s12953-020-00159-3)
Supplement: Supplementary file 1 — Additional file 1. The sequences of identified peptides. [file 12953_2020_159_MOESM1_ESM.docx]

Supplement 1 – The sequences of identified peptides.

#1 Triosephosphate isomerase

| DLGATWVVLGHSER |
| --- |
| LPADTEVVCAPPTAYIDFAR |
| HIFGESDELIGQK |
| VVLAYEPVWAIGTGK |
| VTNGAFTGEISPGMIK |
| IIYGGSVTGATCK |
| CNVSEGVAQCTR |
| IAVAAQNCYK |
| VVFEQTK |
| FFVGGNWK |

#2 Phosphoglycerate mutase

| YADLTEDQLPSCESLK |
| --- |
| FSGWYDADLSPAGHEEAK |
| HGESAWNLENR |
| ALPFWNEEIVPQIK |
| KAMEAVAAQGK |
| AMEAVAAQGK |
| VLIAAHGNSLR |
| SYDVPPPPMEPDHPFYSNISK |
| NLKPIKPMQFLGDEETVR |
| HYGGLTGLNK |
| YADLTEDQLPSCESLKDTIAR |
| HGEAQVK |
| RGGQALR |

#3 Dihydropteridine reductase

| MTDSFTEQADQVTAEVGK |
| --- |
| NSGMPSGAAAIAVLPVTLDTPMNR |
| RPNSGSLIQVVTTDGK |
| VDAILCVAGGWAGGNAK |
| AALDGTPGMIGYGMAK |
| QSIWTSTISSHLATK |
| EGGLLTLAGAK |
| NCDLMWK |

#4 Pyridine nucleotide-disulfide oxidoreductase

| TLGAQLPQYYEVLTAPISK |
| --- |
| CPVQGLYLCGSGAHPGGGVMGAAGR |
| VLDQWFESEPLK |
| VFDCIEAYAPGFK |
| ISQLDTQSPVTK |
| VQGVVLQGGEEVR |
| CPVQGLYLCGSGAHPGGGVMGAAGR |
| KNTYADK |
| NAAHIVFR |
| DILTPQDLER |
| INVAVDR |
| VQVNSEGR |
| VWDEQKK |
| LPNFQAAPNAPGDQPQAHHQCSIHLNCEDTLLLHQAFEDAK |

#5 Phosphotriesterase-related protein

| VLQATAHAQAQLGCPVIIHPGR |
| --- |
| GGGAVVENTTTGLSR |
| CGVIGEIGCSWPLTDSER |
| NPYSHQENLQLNQEVEAVR |
| VQTVLGPVEPSQLGR |
| YGGHGYSHILTNVVPK |
| VLQEAGADISK |
| FLVNEGYEDR |
| NPGAPFQIIR |
| TVMSHLDR |
| NLFWIQK |
| ILMAHDIHTK |

#6 Aminoacylase-1A

| QLQSWCQEAGEGVTFEFAQK |
| --- |
| ICTVQPNPDYGSAVTFLEER |
| AGFALDEGLANPTDAFTVFYSER |
| MTPTDDTDPWWAAFSGACK |
| EMNLTLEPEIFPAATDSR |
| LEGGVAYNVVPATMSACFDFR |
| EMNLTLEPEIFPAATDSR |
| TIHMTFVPDEEVGGHK |
| AVGIPALGFSPMNR |
| TIHMTFVPDEEVGGHK |
| SVSIQYLEAVR |
| GPESEHPSVTLFR |
| RPEFQALR |
| VVNSILAFR |
| DSEGYIYAR |
| EGAVTSVNLTK |
| TPVLLHDHNER |
| VTSTGKPGHASR |
| LVAALASVPALPGES |
| LHEAVFLR |
| EHWHHDPFEAFK |
| LQANPHLK |
| FIEDTAAEK |
| GMELFVK |
| GVDIYTR |
| GMELFVK |
| SPWWIR |
| QLGLSCQK |
| VAPDVDMK |

#7 N(G),N(G)-dimethylarginine dimethylaminohydrolase 1

| DENATLDGGDVLFTGR |
| --- |
| QHQLYVGVLGSK |
| DYAVSTVPVADSLHLK |
| LQLNIVEMK |
| GAEILADTFK |
| SQGEEVDFAR |
| IMQQMSDHR |
| ATHAVVR |

#8 Phosphoglycerate kinase 1

| TGQATVASGIPAGWMGLDCGTESSK |
| --- |
| GCITIIGGGDTATCCAK |
| VSHVSTGGGASLELLEGK |
| VLNNMEIGTSLYDEEGAK |
| AHSSMVGVNLPQK |
| LGDVYVNDAFGTAHR |
| ALESPERPFLAILGGAK |
| YSLEPVAAELK |
| IQLINNMLDK |
| VLPGVDALSNV |
| ITLPVDFVTADK |
| KYAEAVAR |
| AGGFLMK |
| KELNYFAK |
| FHVEEEGK |
| SLMDEVVK |
| YAEAVAR |

| **Name of protein** | **List of peptides** |
| --- | --- |
| #1 Triosephosphate isomerase | DLGATWVVLGHSER |
|  | LPADTEVVCAPPTAYIDFAR |
|  | HIFGESDELIGQK |
|  | VVLAYEPVWAIGTGK |
|  | VTNGAFTGEISPGMIK |
|  | IIYGGSVTGATCK |
|  | CNVSEGVAQCTR |
|  | IAVAAQNCYK |
|  | VVFEQTK |
|  | FFVGGNWK |
| #2 Phosphoglycerate mutase | YADLTEDQLPSCESLK |
|  | FSGWYDADLSPAGHEEAK |
|  | HGESAWNLENR |
|  | ALPFWNEEIVPQIK |
|  | KAMEAVAAQGK |
|  | AMEAVAAQGK |
|  | VLIAAHGNSLR |
|  | SYDVPPPPMEPDHPFYSNISK |
|  | NLKPIKPMQFLGDEETVR |
|  | HYGGLTGLNK |
|  | YADLTEDQLPSCESLKDTIAR |
|  | HGEAQVK |
|  | RGGQALR |
| #3 Dihydropteridine reductase | MTDSFTEQADQVTAEVGK |
|  | NSGMPSGAAAIAVLPVTLDTPMNR |
|  | RPNSGSLIQVVTTDGK |
|  | VDAILCVAGGWAGGNAK |
|  | AALDGTPGMIGYGMAK |
|  | QSIWTSTISSHLATK |
|  | EGGLLTLAGAK |
|  | NCDLMWK |
| #4 Pyridine nucleotide-disulfide oxidoreductase | TLGAQLPQYYEVLTAPISK |
|  | CPVQGLYLCGSGAHPGGGVMGAAGR |
|  | VLDQWFESEPLK |
|  | VFDCIEAYAPGFK |
|  | ISQLDTQSPVTK |
|  | VQGVVLQGGEEVR |
|  | CPVQGLYLCGSGAHPGGGVMGAAGR |
|  | KNTYADK |
|  | NAAHIVFR |
|  | DILTPQDLER |
|  | INVAVDR |
|  | VQVNSEGR |
|  | VWDEQKK |
|  | LPNFQAAPNAPGDQPQAHHQCSIHLNCEDTLLLHQAFEDAK |
| #5 Phosphotriesterase-related protein | VLQATAHAQAQLGCPVIIHPGR |
|  | GGGAVVENTTTGLSR |
|  | CGVIGEIGCSWPLTDSER |
|  | NPYSHQENLQLNQEVEAVR |
|  | VQTVLGPVEPSQLGR |
|  | YGGHGYSHILTNVVPK |
|  | VLQEAGADI |
|  | FLVNEGYEDR |
|  | NPGAPFQIIR |
|  | TVMSHLDR |
|  | NLFWIQK |
|  | ILMAHDIHTK |
| #6 Aminoacylase-1A | QLQSWCQEAGEGVTFEFAQK |
|  | ICTVQPNPDYGSAVTFLEER |
|  | AGFALDEGLANPTDAFTVFYSER |
|  | MTPTDDTDPWWAAFSGACK |
|  | EMNLTLEPEIFPAATDSR |
|  | LEGGVAYNVVPATMSACFDFR |
|  | EMNLTLEPEIFPAATDSR |
|  | TIHMTFVPDEEVGGHK |
|  | AVGIPALGFSPMNR |
|  | TIHMTFVPDEEVGGHK |
|  | SVSIQYLEAVR |
|  | GPESEHPSVTLFR |
|  | RPEFQALR |
|  | VVNSILAFR |
|  | DSEGYIYAR |
|  | EGAVTSVNLTK |
|  | TPVLLHDHNER |
|  | VTSTGKPGHASR |
|  | LVAALASVPALPGES |
|  | LHEAVFLR |
|  | EHWHHDPFEAFK |
|  | LQANPHLK |
|  | FIEDTAAEK |
|  | GMELFVK |
|  | GVDIYTR |
|  | GMELFVK |
|  | SPWWIR |
|  | QLGLSCQK |
|  | VAPDVDMK |
| #7 N(G),N(G)-dimethylarginine dimethylaminohydrolase 1 | DENATLDGGDVLFTGR |
|  | QHQLYVGVLGSK |
|  | DYAVSTVPVADSLHLK |
|  | LQLNIVEMK |
|  | GAEILADTFK |
|  | SQGEEVDFAR |
|  | IMQQMSDHR |
|  | ATHAVVR |
| #8 Phosphoglycerate kinase 1 | TGQATVASGIPAGWMGLDCGTESSK |
|  | GCITIIGGGDTATCCAK |
|  | VSHVSTGGGASLELLEGK |
|  | VLNNMEIGTSLYDEEGAK |
|  | AHSSMVGVNLPQK |
|  | LGDVYVNDAFGTAHR |
|  | ALESPERPFLAILGGAK |
|  | YSLEPVAAELK |
|  | IQLINNMLDK |
|  | VLPGVDALSNV |
|  | ITLPVDFVTADK |
|  | KYAEAVAR |
|  | AGGFLMK |
|  | KELNYFAK |
|  | FHVEEEGK |
|  | SLMDEVVK |
|  | YAEAVAR |
