# Supplementary material for: Protection of the transplant kidney during cold perfusion with doxycycline: proteomic analysis in a rat model
Source: Proteome Sci. 2020 Apr 20;18:3. doi: 10.1186/s12953-020-00159-3 (PMC7171734; doi:10.1186/s12953-020-00159-3)

Supplement 2 – Two-Dimensional electrophoresis (2-DE) gel images.

Gels #1-4: Control group

Gel #1


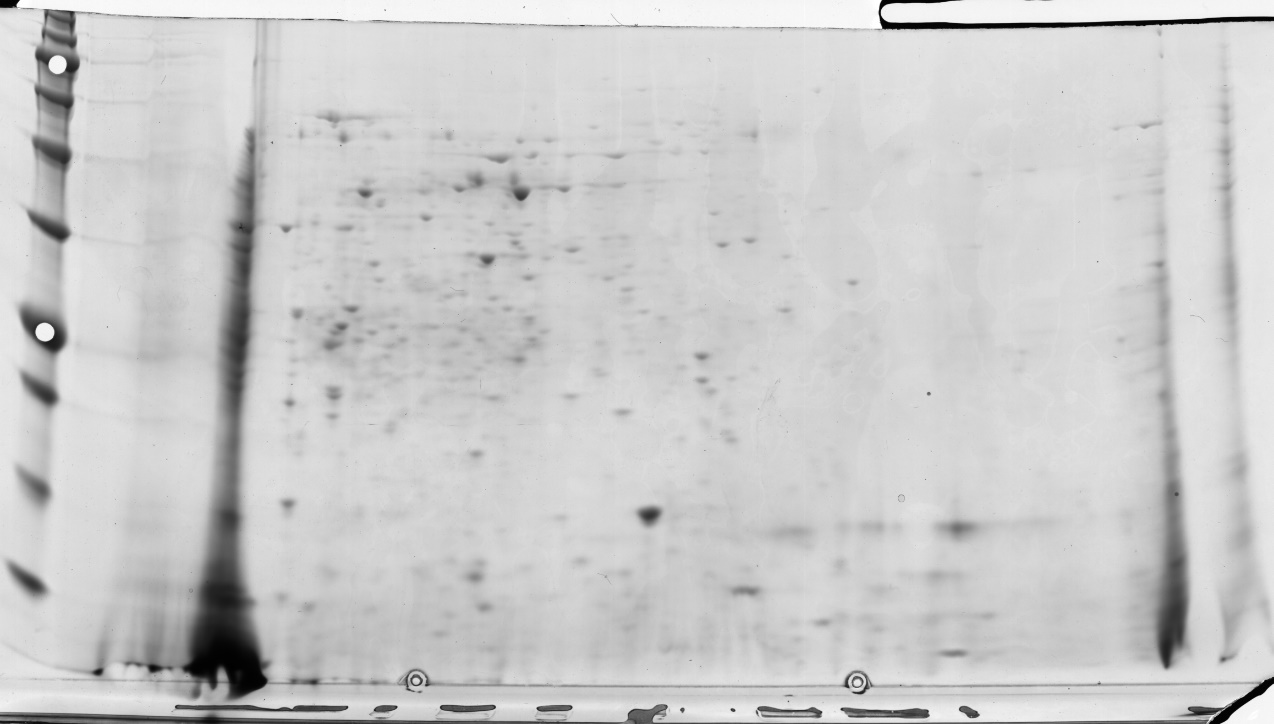


Gel #2


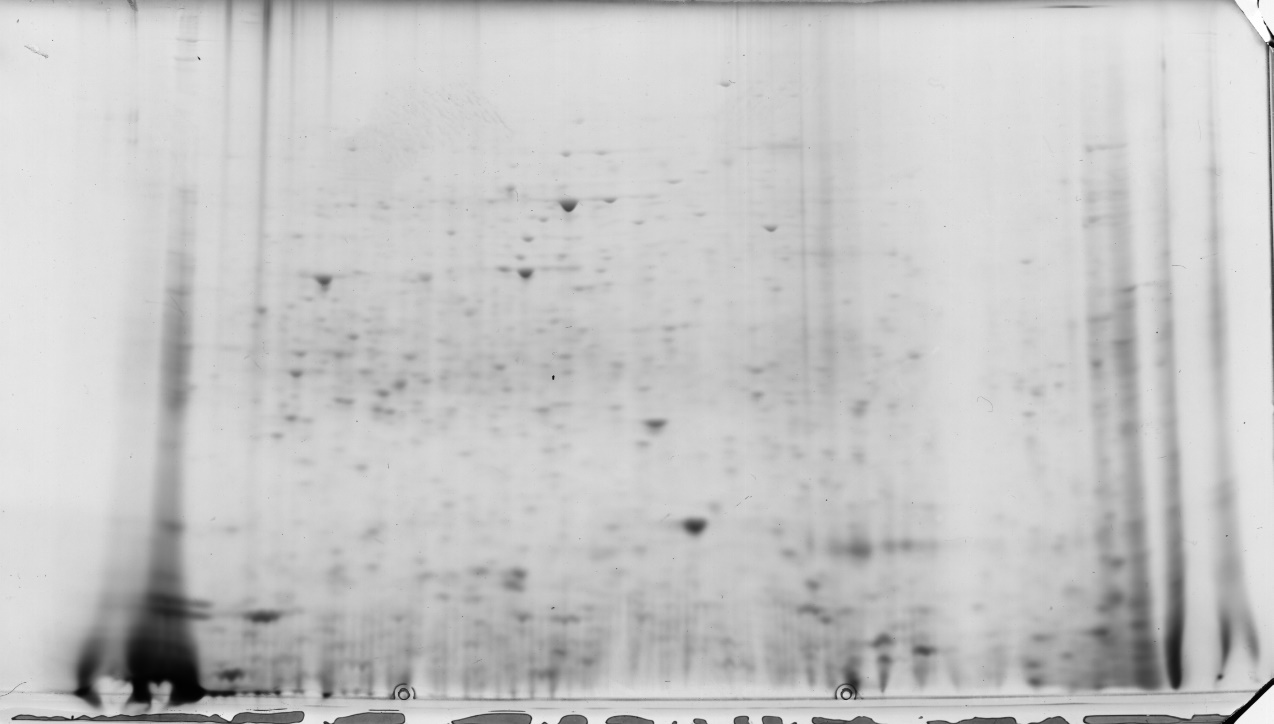


Gel #3


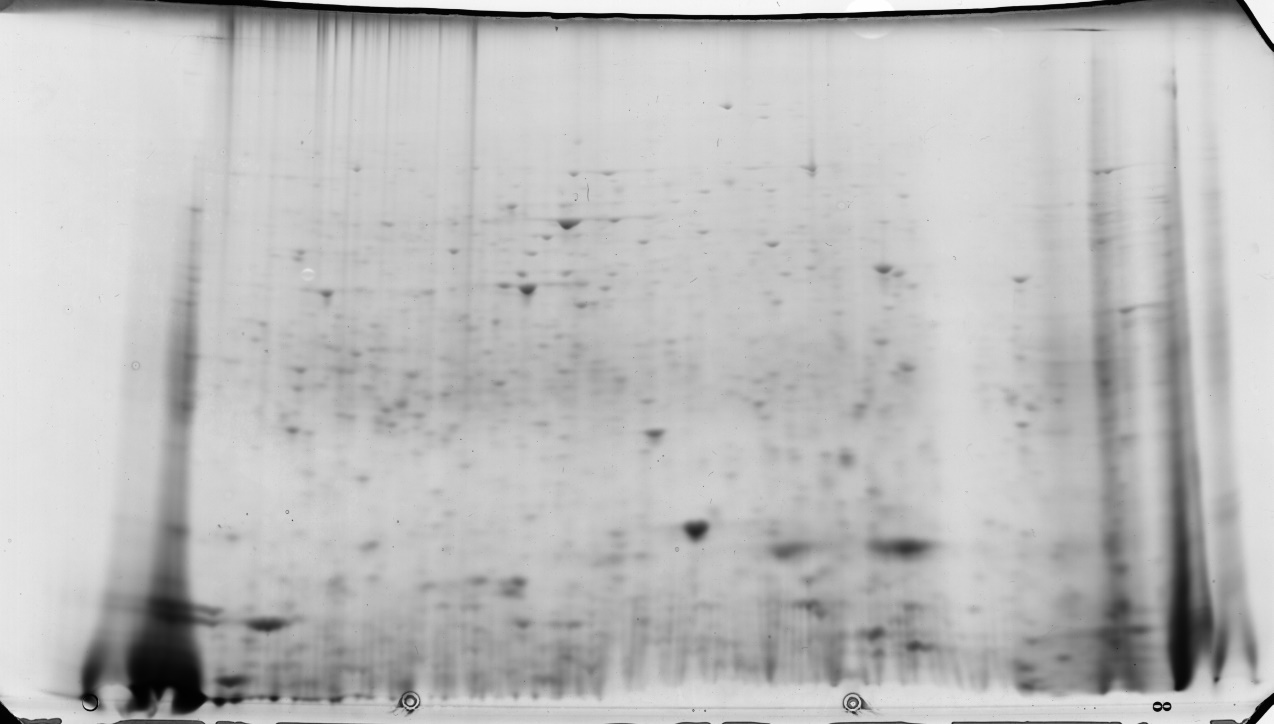


Gel #4


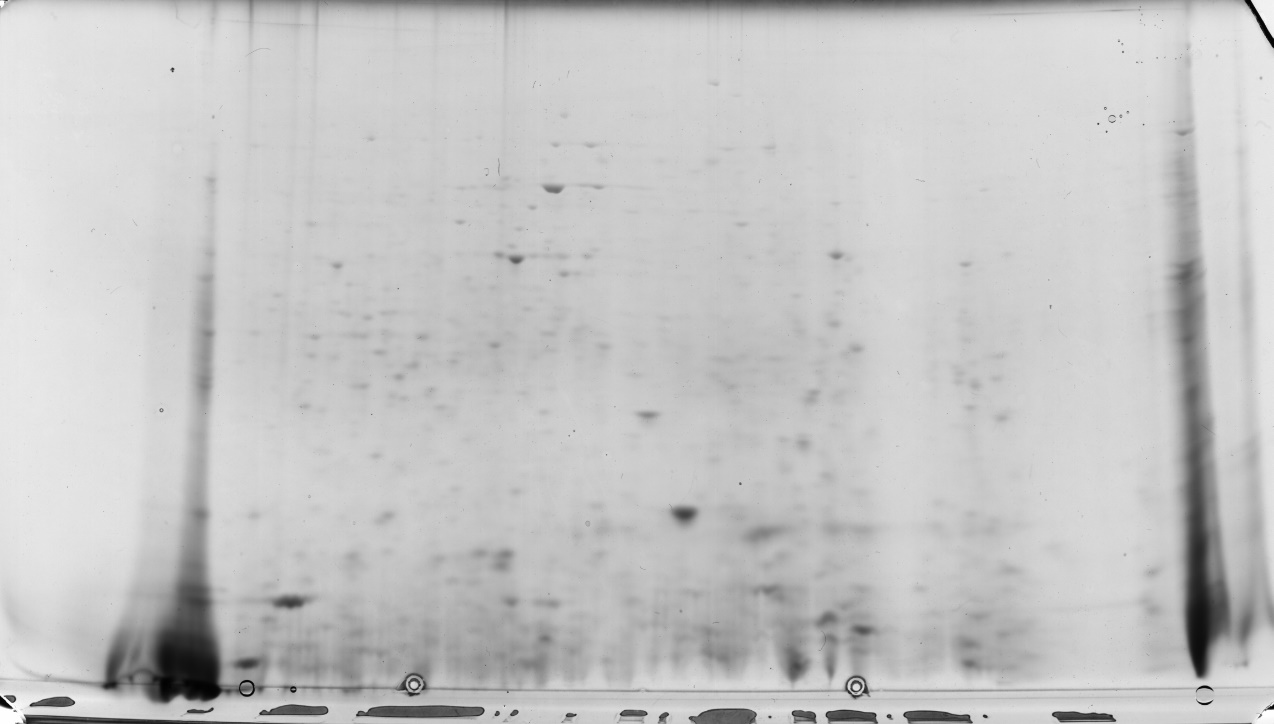


Gels #5-8: 22h of cold perfusion

Gel #5


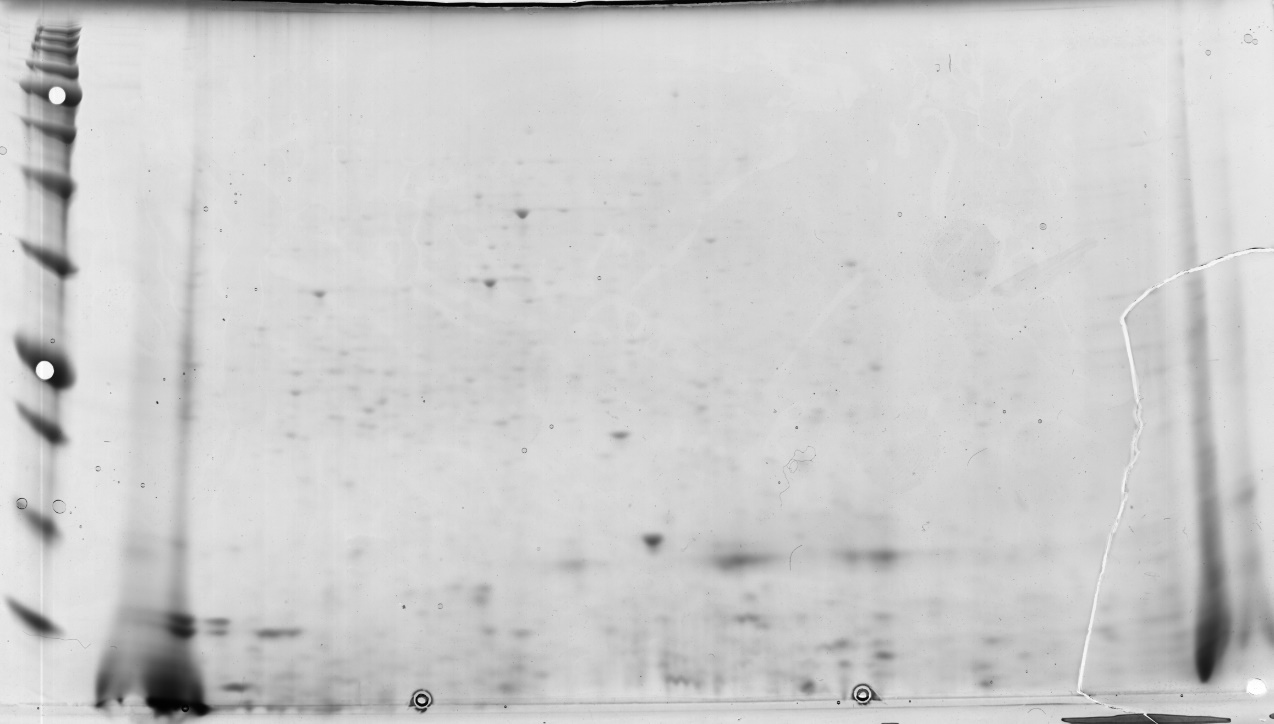


Gel #6


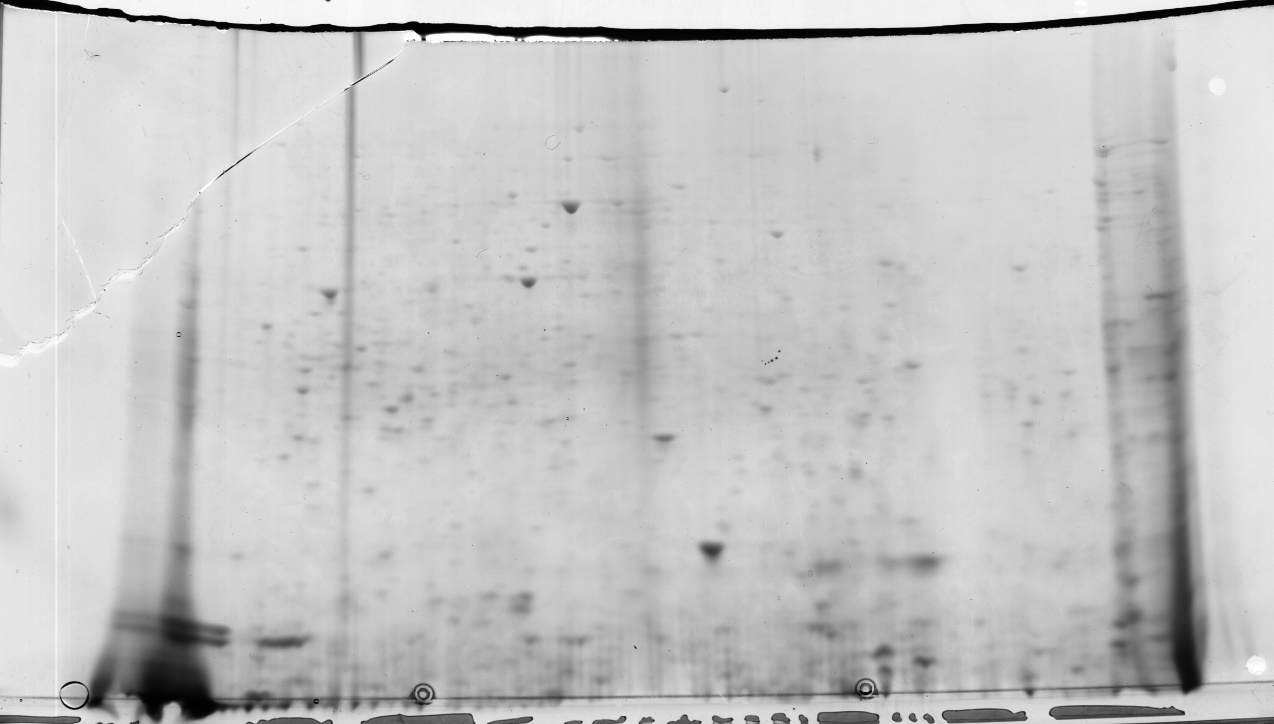


Gel #7


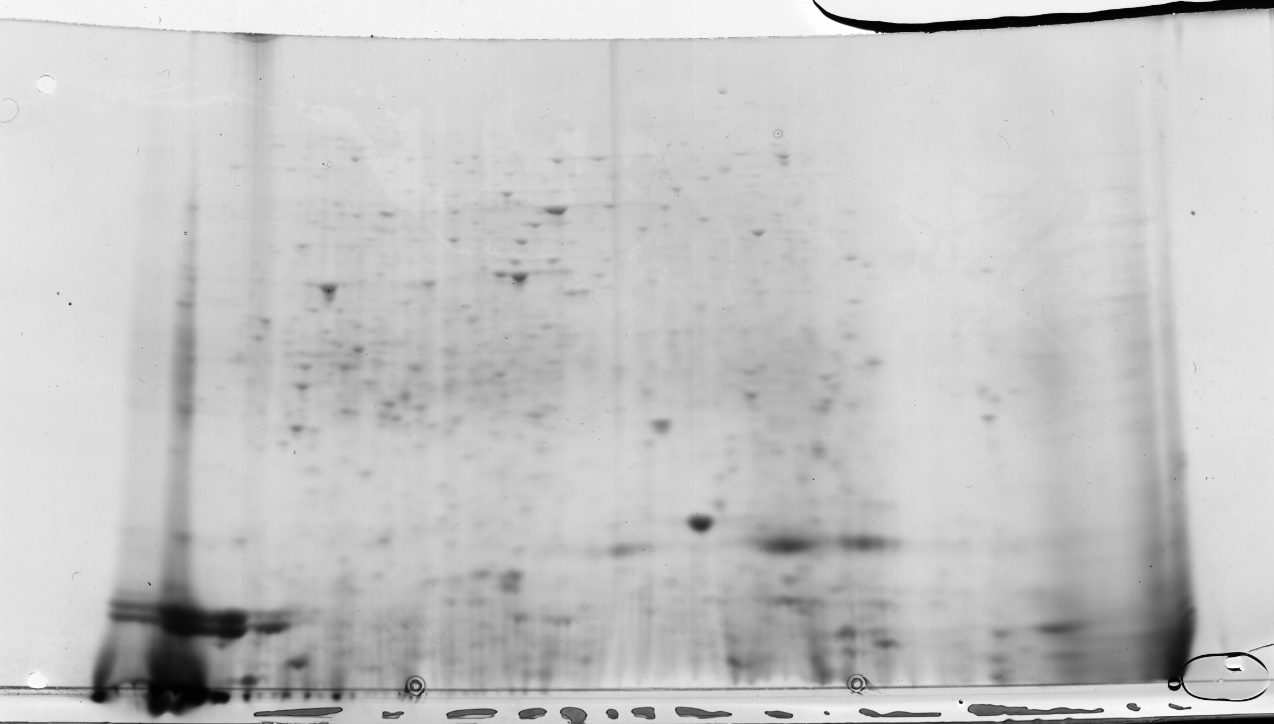


Gel #8


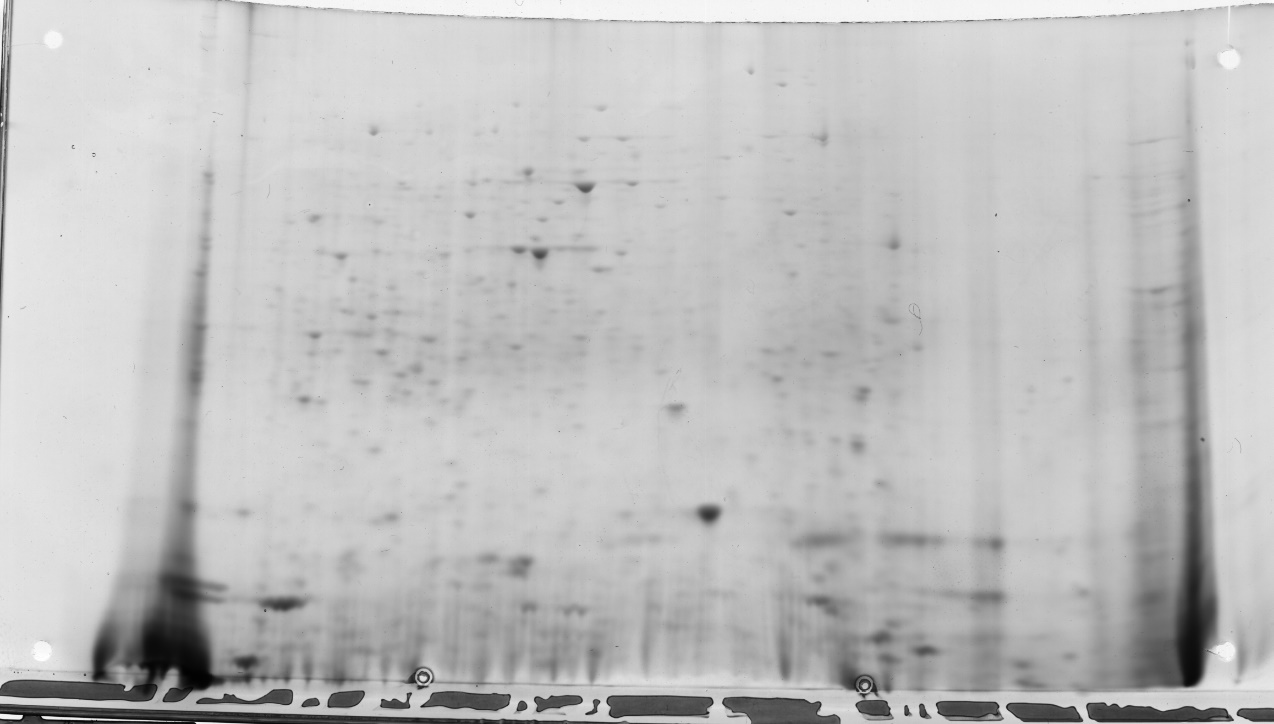


Gels #9-12: 22h of cold perfusion with doxycycline

Gel #9


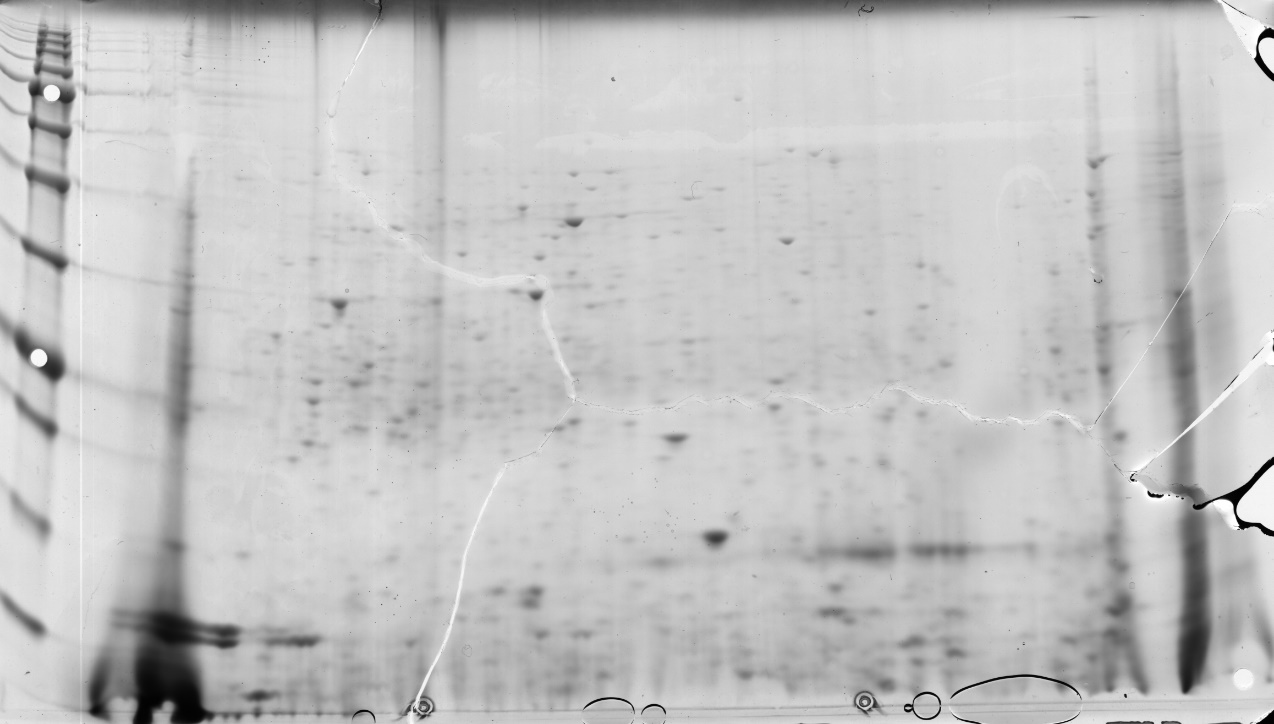


Gel #10


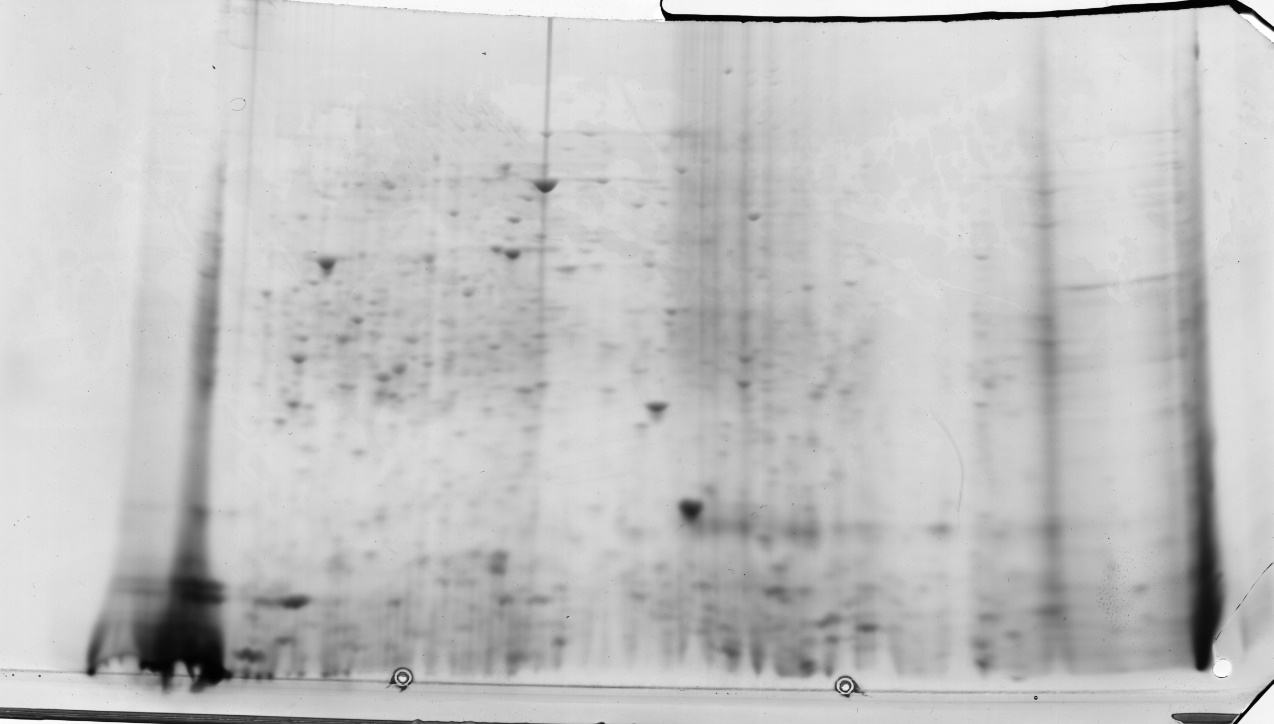


Gel #11


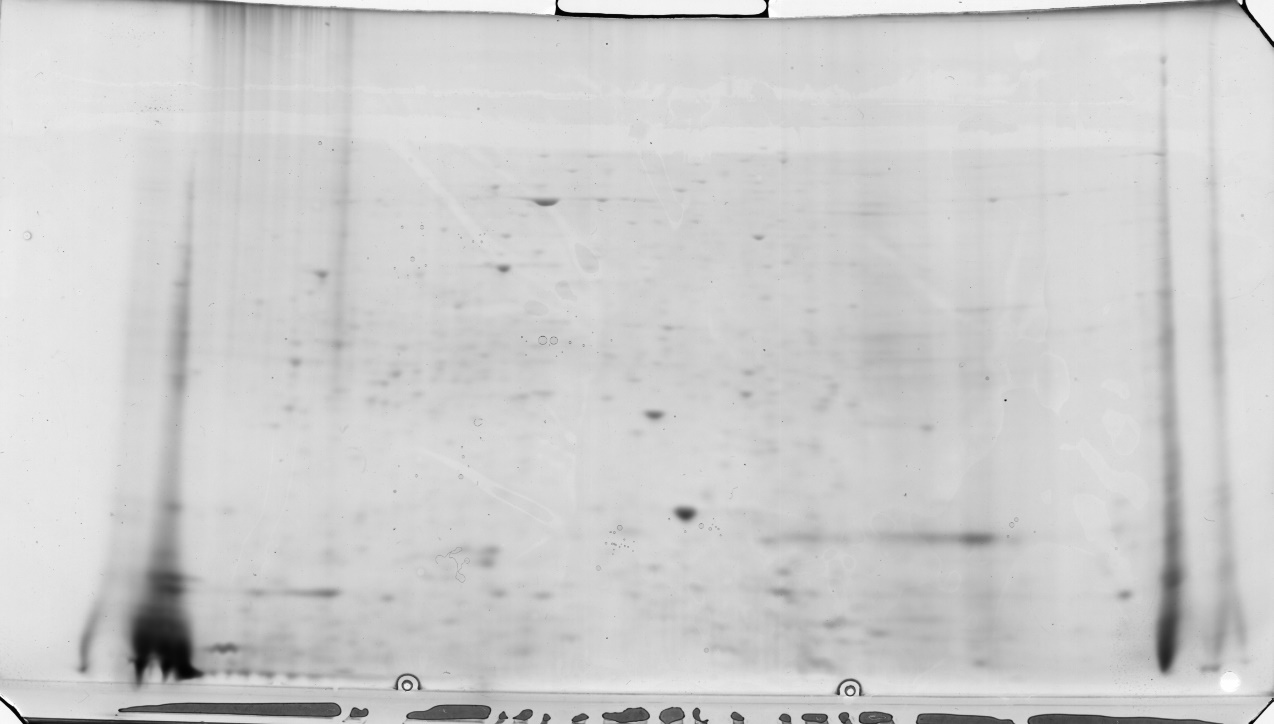


Gel #12


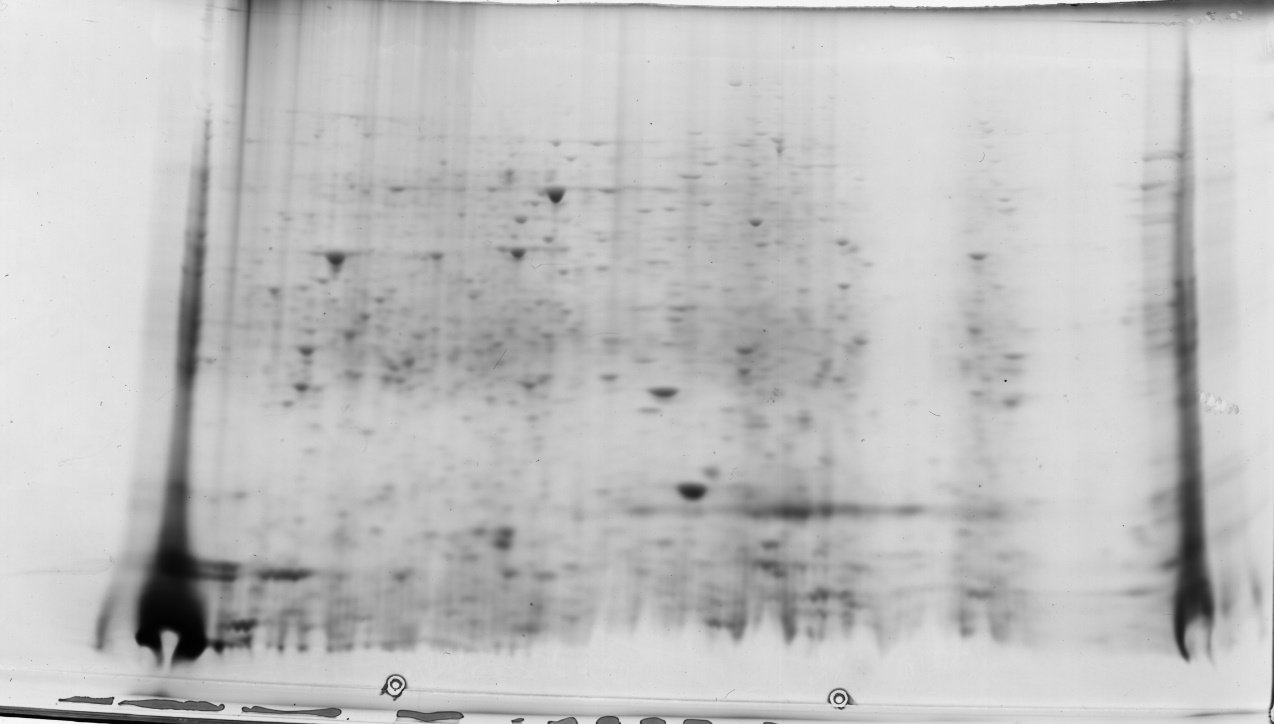

Supplement: Supplementary file 2 — Additional file 2. Two-dimensional gel electrophoresis gel images. [file 12953_2020_159_MOESM2_ESM.docx]
