# Supplementary material for: Protection of the transplant kidney during cold perfusion with doxycycline: proteomic analysis in a rat model
Source: Proteome Sci. 2020 Apr 20;18:3. doi: 10.1186/s12953-020-00159-3 (PMC7171734; doi:10.1186/s12953-020-00159-3)
Supplement: Supplementary file 3 — Additional file 3. Western blot images [file 12953_2020_159_MOESM3_ESM.pptx]

## Slide 1
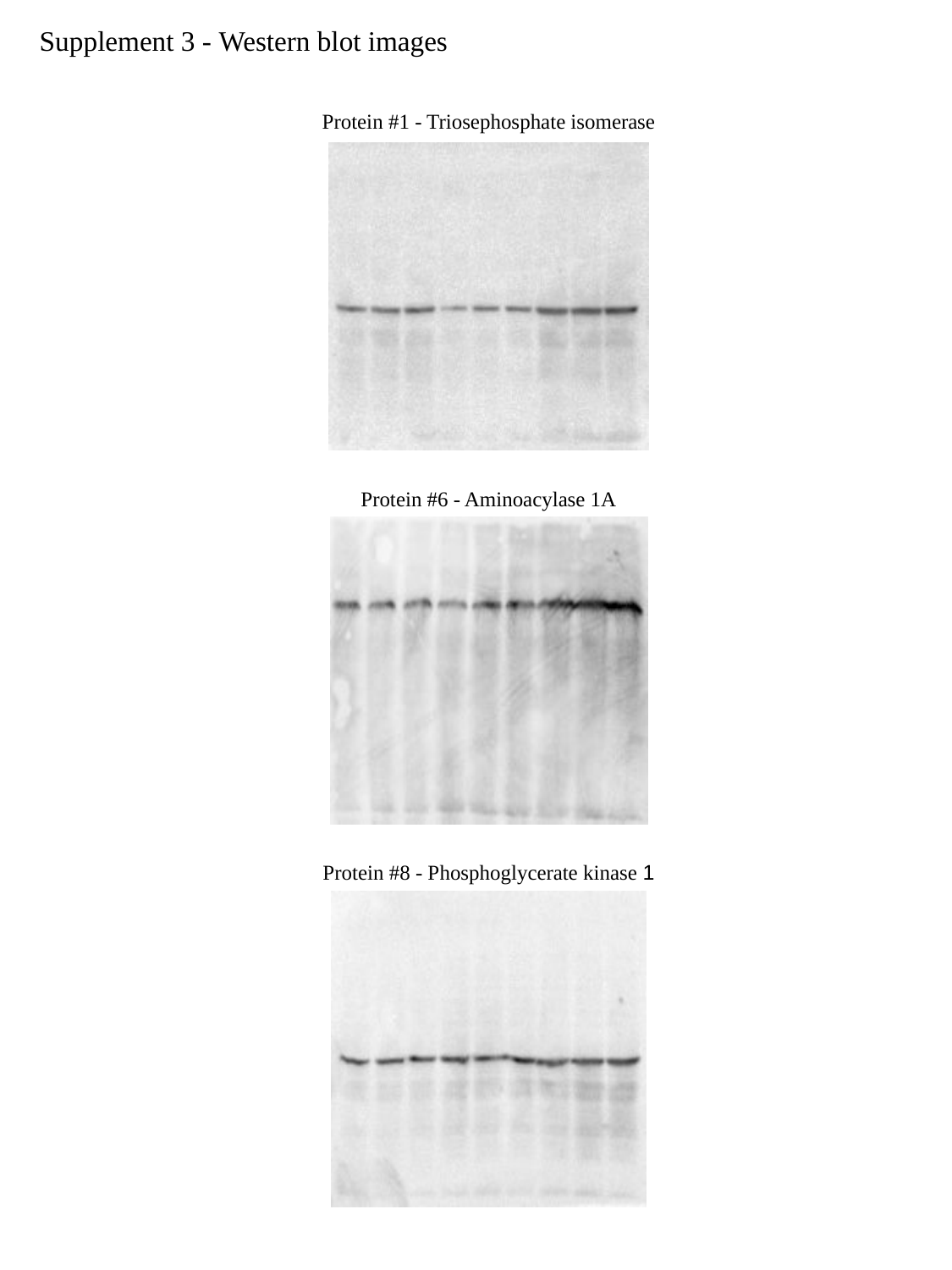

Supplement 3 - Western blot images
Protein #1 - Triosephosphate isomerase
Protein #6 - Aminoacylase 1A
Protein #8 - Phosphoglycerate kinase 1
