# Supplementary material for: Protection of the transplant kidney during cold perfusion with doxycycline: proteomic analysis in a rat model
Source: Proteome Sci. 2020 Apr 20;18:3. doi: 10.1186/s12953-020-00159-3 (PMC7171734; doi:10.1186/s12953-020-00159-3)
Supplement: Supplementary file 4 — Additional file 4. Electron microscopy [file 12953_2020_159_MOESM4_ESM.pptx]

## Slide 1
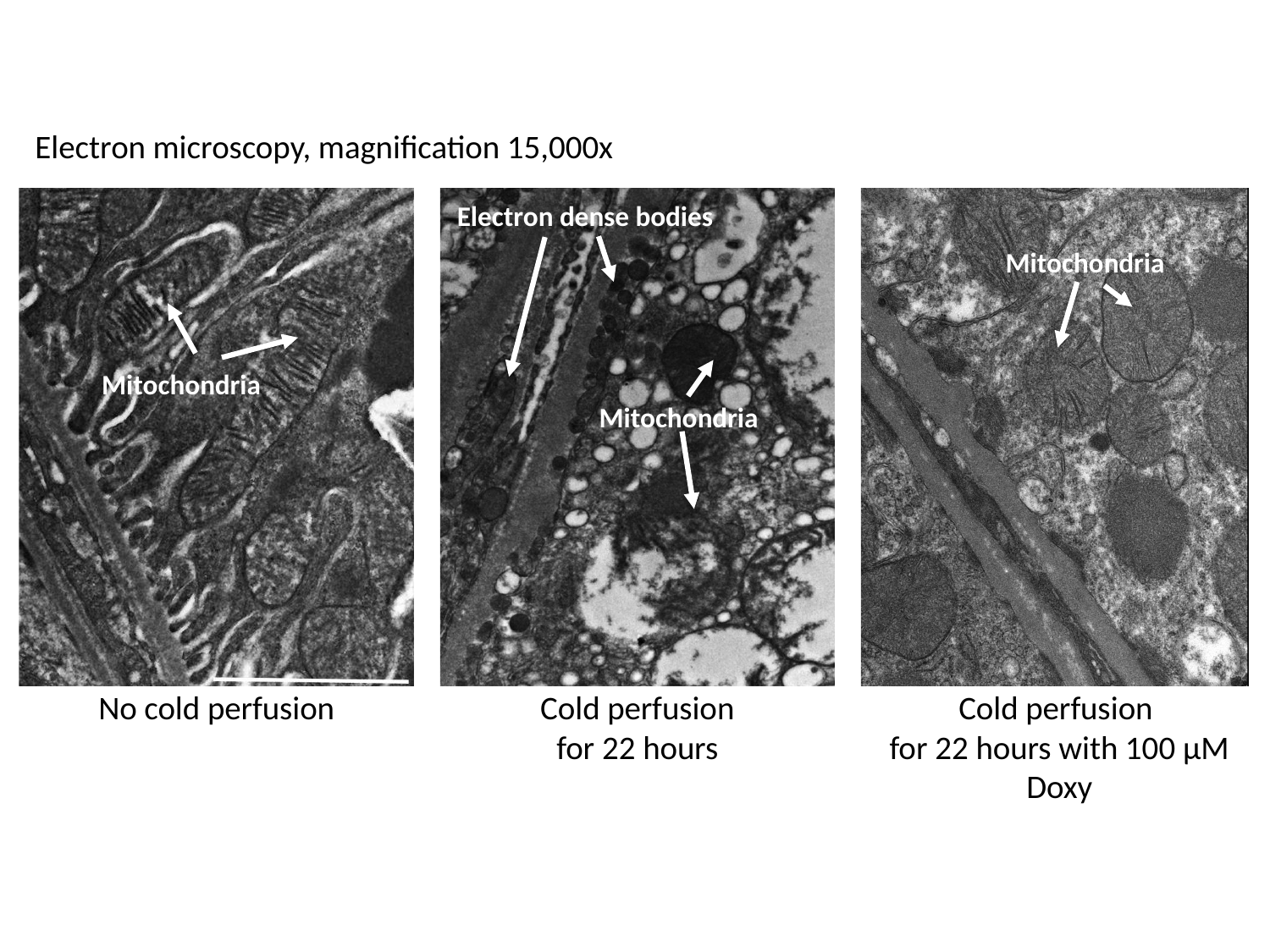

Electron microscopy, magnification 15,000x
Electron dense bodies
Mitochondria
Mitochondria
Mitochondria
No cold perfusion
Cold perfusion
for 22 hours
Cold perfusion
for 22 hours with 100 µM Doxy
